# Allostatic Load, Social Participation, and Healthy Ageing: Longitudinal Evidence on the Impact of Chronic Stress

**DOI:** 10.3390/geriatrics10060157

**Published:** 2025-11-27

**Authors:** Lujain Sahab, Jonathon Timothy Newton, Wael Sabbah

**Affiliations:** Faculty of Dentistry, Oral & Craniofacial Sciences, King’s College London, Bessemer Road, Denmark Hill, London SE5 9RS, UK

**Keywords:** healthy ageing, allostatic load, social participation, older adults, Health and Retirement Study

## Abstract

**Background/Objectives**: The study aimed to examine the impact of allostatic load on healthy ageing over a decade and whether social participation attenuates this relationship among older American adults. **Methods**: Data were extracted from three waves (wave 8, wave 10, wave 13) of the Health and Retirement Study, a longitudinal survey of American adults. The analysis included allostatic load, socioeconomic (education) and demographic (gender, age, ethnicity, and marital status) factors at baseline, social participation in wave 10, and healthy ageing in wave 10 and wave 13. A latent variable was created for allostatic load that included waist circumference, C-reactive protein, glycated hemoglobin, and blood pressure. Healthy ageing was defined as an aggregate measure including freedom from disability, freedom from cognitive impairment, and high physical functioning. Social participation was a dichotomous variable that included individuals’ work status, perceived neighbourhood safety, and partaking in volunteer work. Structural equation modelling was used to examine the direct and indirect relationships between these factors and healthy ageing. **Results**: A total of 14,537 participants with complete data in all waves were included in the analysis. The mean age at baseline was 68.7 years. Results showed a significant association between higher allostatic load and lower healthy ageing (estimate = −0.12, 95% CI: −0.14, −0.11). Allostatic load was negatively associated with social participation (estimate = −0.32, 95% CI: −0.34, −0.30). Social participation showed a positive significant association with healthy ageing, indicating partial buffering that accounted for 12% of the total effect. Higher educational attainment was associated with better healthy-ageing outcomes, whereas non-Black ethnicity was linked to poorer healthy ageing. **Conclusions**: Elevated allostatic load was associated with poorer ageing outcomes, with social participation partially attenuating the relationship. Higher education predicted more favourable trajectories, while ethnic differences suggested resilience among older Black adults. These results indicate that both physiological and social factors contribute to variations in healthy ageing.

## 1. Introduction

The pursuit of healthy ageing, defined as the process of developing and maintaining the functional ability that enables well-being in older age [1], has become a central focus within neuroscience, behavioural medicine, and public health [2]. Although ageing is shaped by a complex interaction of biological, behavioural, and environmental influences, an increasing number of studies highlight chronic psychosocial stress as a significant and potentially modifiable factor that contributes to accelerated biological ageing. Prolonged exposure to stress disrupts internal physiological balance and results in cumulative biological strain, a process conceptualized as allostatic load [3].

Allostatic load was first introduced by McEwen and Stellar [3] to describe the cumulative “wear and tear” on the body and brain resulting from recurrent or chronic stress. While homeostasis signifies the maintenance of internal stability, allostasis describes the active process through which the body adapts to external challenges. Although allostasis is essential for short term survival and adaptation, its repeated or prolonged activation can lead to physiological dysregulation. Allostatic load captures the long-term cost of this adaptation and is typically measured through biomarkers reflecting dysfunction in the neuroendocrine, immune, cardiovascular, and metabolic systems [4,5].

Recent evidence suggests that elevated allostatic load is associated with a variety of negative outcomes in brain and behavioural health [6,7,8]. Beyond neurological outcomes, higher allostatic load is linked to various adverse health outcomes across longitudinal population studies, including increased risk of chronic pain, functional limitations, frailty, chronic conditions, and premature mortality [9,10,11].

While allostatic load remains a critical determinant of ageing, increasing attention is being directed towards the role of social participation as a potential buffer against its detrimental effects. Social participation, encompassing activities such as employment, volunteering, and living in a safe and supportive neighbourhood, has been linked with improved mental and physical health and lower stress levels [12]. A well-established mechanism through which social support influences health is the “stress-buffering” effect, whereby supportive social relationships help individuals cope more effectively with stressors and attenuate their negative impact on health outcomes [13]. Empirical evidence supports this pathway, showing that individuals with higher levels of social support experience fewer adverse physiological and psychological effects in response to stress [14].

Allostatic load offers a valuable framework for understanding how chronic stress becomes biologically embedded and undermines healthy ageing. Although growing evidence suggests that social participation may buffer the adverse physiological effects of chronic stress on healthy ageing, few studies have examined this mediating role within a longitudinal framework. This study builds on the stress-buffering theory and a multidimensional model of healthy ageing by investigating how allostatic load directly influences ageing outcomes and whether social participation attenuates that relationship.

## 2. Materials and Methods

### 2.1. Data Source

Data were drawn from the Health and Retirement Study (HRS), a longitudinal, nationally representative survey of Americans aged 50 years and older. Data were extracted from three biennial waves: wave 8 (2006), wave 10 (2010), and wave 13 (2016).

### 2.2. Study Design and Sample

The HRS follows a large cohort of adults across the United States, collecting information on health, demographics, and socioeconomic factors. For this analysis, the final sample comprised 14,537 participants, with repeated measures drawn from the three selected waves.

### 2.3. Study Variables

This analysis included an extensive range of predictor variables, organized into the following categories. Allostatic load, social participation, demographic and socioeconomic factors. Allostatic load, demographic and socioeconomic factors were all collected from wave 8 in 2006.

Demographic factors comprise gender (male/female); age (measured as a continuous variable); marital status, grouped into married, unmarried/divorced, or widowed; and race/ethnicity, categorized as non-Black Americans and Black Americans. Socioeconomic factors included education, which was classified into four stages: <high school, high school diploma, some college, and college degree or higher.

Allostatic load, operationalized through biomarkers of chronic stress, reflects cumulative physiological dysregulation across multiple systems [3,5]. In this study allostatic load was constructed using five biomarkers representing key physiological systems known to be affected by chronic stress: waist circumference, C-reactive protein (CRP), high-density lipoprotein (HDL), glycated hemoglobin (HbA1c), and high blood pressure. Variables were categorized based on recognized cut off points. Waist circumference was considered as normal ≤ 39 inches, high ≥ 40 inches for men, and normal ≤ 34 inches, high ≥ 35 inches for women [15]. CRP was documented as normal < 3 mg/L, high ≥ 3 mg/L [16]. HDL was classified as normal <40 mg/dL, high ≥40 mg/dL for men, and normal < 50 mg/dL, high ≥ 50 mg/dL for women [17]. HbA1c was documented as normal < 6.5%, diabetic ≥ 6.5% [18]. High blood pressure was specified by systolic ≥ 140 mm Hg and diastolic ≥ 90 mm Hg for both genders [19]. The measure was validated in a previous study [11].

Social participation, from wave 10-2010, was defined as a multidimensional concept, incorporating three primary components identified in earlier research [20]. These components were employment status, perception of neighbourhood safety, and engagement in volunteer activities. Participants were asked whether they were currently employed for pay (0 = no, 1 = yes), how safe they considered their neighbourhood (fair/poor = 0, good/very good/excellent = 1), and whether they had participated in volunteer work during the previous 12 months for religious, educational, health related, or other charitable organizations (yes/no). Social participation was dichotomized (yes/no), with individuals meeting all three criteria classified as socially active.

The outcome variable, healthy ageing, was collected at two points, wave 10-2010 and wave 13-2016, and operationalized as a composite measure, incorporating freedom from cognitive function, freedom from disability, and high physical functioning. This approach is consistent with the World Health Organization’s (WHO) Decade of Healthy Ageing (2021–2030), which highlights the significance of maintaining functional abilities and overall well-being in later life [21]. The measure was constructed following previously validated methods. For analysis, healthy ageing was coded as 1 = healthy, representing individuals who were healthy across all three domains, and 0 = not healthy [22]. An additional variable for health ageing from wave 10 (2010) was also created to be included in the analytical model.

### 2.4. Statistical Analysis

The distributions of all variables incorporated in the analysis from each wave were assessed. Structural equation modelling (SEM) was used to test the associations. The analysis was performed in Mplus software Version 8.11 (Muthén and Muthén, 1998–2017) with maximum likelihood estimation. Model fit was evaluated using the chi-square statistic (χ^2^), comparative fit index (CFI), Tucker–Lewis index (TLI), and root mean square error of approximation (RMSEA).

Two models were developed using SEM to study the complex relationships between allostatic load, social participation, and healthy ageing. The analysis was adjusted for socioeconomic and demographic factors. The first path assessed the direct effect of allostatic load (latent variable) on healthy ageing. The analysis incorporated one measure of socioeconomic factors (education) and demographic variables (age, gender, ethnicity, marital status) as covariates to control for potential confounding influences. The goal was to establish whether higher allostatic load levels were associated with poorer healthy-ageing outcomes. The second (indirect) pathway included social participation as a buffer, as a means of exploring whether it attenuates the relationship between allostatic load and healthy ageing. Healthy ageing in 2010 was also included in the model as a covariate to account for change over time.

## 3. Results

### 3.1. Characteristics of the Study Sample

**Figure 1:**
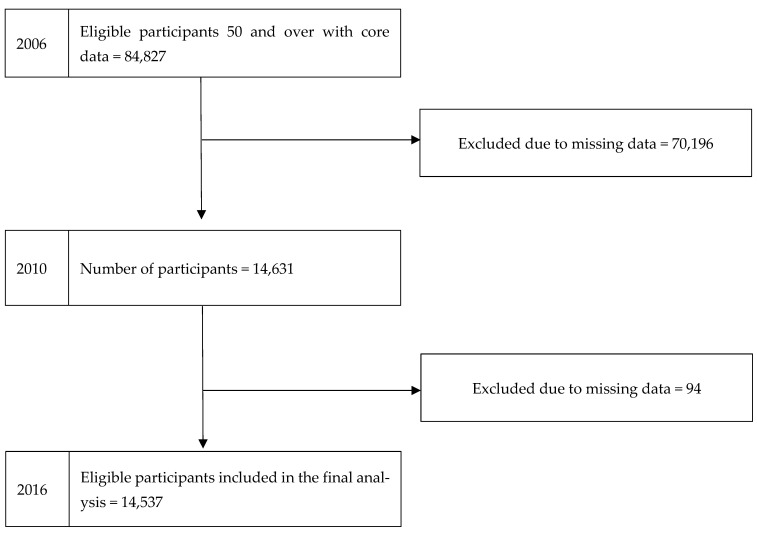
Flowchart showing the number of participants included in the analysis (HRS-USA 2006–2010–2016).

The study sample comprised 14,537 individuals with complete data in every wave. Figure 1 shows a flowchart representing participant numbers throughout the study and reasons for attrition. Most of the participants were female and of non-Black ethnicity. The baseline sample had a mean age of 68.7 years; 28.5% had completed education at the college level or above. Among individuals aged 50 years and older, the proportion achieving healthy ageing at the final follow-up was 50.3%, representing a decline from 53.8% in 2010. This means half of the participants achieved healthy ageing at final follow-up. In 2010, 78.3% of participants reported engagement in social participation. Table 1 provides a detailed overview of all study variables.

### 3.2. Pathways Linking Allostatic Load to Healthy Ageing

This study utilized structural equation modelling to test the direct connection between allostatic load and healthy ageing, and the indirect relationship by using social participation as a buffer (Table 2). The latent construct of allostatic load in 2006 was robustly defined by its physiological indicators. All indicators were statistically significant, supporting the validity of the latent allostatic load construct.

The results indicated that higher allostatic load in 2006 was directly associated with lower healthy ageing in 2016 (estimate = −0.12, 95% CI: −0.14, −0.11) allostatic load was also negatively associated with social participation in 2010 (estimate = −0.32, 95% CI: −0.34, −0.30), which in turn positively predicted healthy ageing (estimate = 0.05, 95% CI: 0.04, 0.06). The resulting indirect effect of allostatic load on healthy ageing via social participation was −0.016 (*p* < 0.001), accounting for approximately 12% of the total effect (total effect estimate = −0.136). These findings suggest that although social participation partially attenuated the detrimental impact of chronic stress on ageing outcomes, allostatic load continued to exert a substantial direct negative effect (Table 2).

Several covariates were also associated with healthy ageing. Higher levels of education (estimate = 0.18, 95% CI: 0.17, 0.19) and older age (estimate = 0.36, 95% CI: 0.35, 0.37) predicted better healthy ageing. In contrast, belonging to a non-Black ethnic group was associated with poorer healthy ageing (estimate = −0.11, 95% CI: −0.12, −0.10). Gender and marital status were not significantly related to healthy ageing. In addition, healthy ageing in 2010 was strongly predictive of healthy ageing in 2016 (estimate = 0.10, 95% CI: 0.08, 0.12).

**Figure 2:**
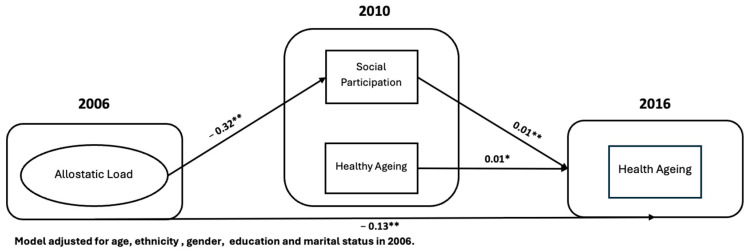
Path diagram presenting the pathways that affect healthy ageing (HRS-USA 2006–2010-2016) (*n* = 14,537). * *p* < 0.05, ** *p* < 0.001.

Figure 2 demonstrates the longitudinal associations between allostatic load, social participation, and healthy ageing in 2016, highlighting both the direct and indirect pathways influencing healthy-ageing outcomes.

## 4. Discussion

This study explored the longitudinal relationship between allostatic load and healthy ageing through structural equation modelling, emphasizing the potential contribution of social participation. The results showed that allostatic load negatively affects healthy ageing over time, confirming the hypothesis of the study. Furthermore, social participation appeared to buffer this relationship, although it remained significant.

The results confirmed that higher allostatic load was consistently associated with lower healthy-ageing scores, highlighting the long-term detrimental impact of chronic physiological stress on ageing outcomes. This finding aligns with the conceptualization of allostatic load as a cumulative measure of physiological wear and tear due to chronic stress, suggesting that elevated biomarkers of stress have a lasting negative impact on ageing outcomes [5,23,24]

Importantly, social participation in 2010 reduced the negative impact of allostatic load on healthy ageing in 2016. Higher allostatic load was associated with lower social engagement; however, higher social participation predicted better healthy-ageing outcomes. The indirect effect accounted for approximately 12% of the total effect. This indicates that social participation can buffer some of the negative consequences of chronic stress, supporting the view that social engagement is a protective factor in later-life health. This suggests that while enhancing social participation may mitigate some of the impacts of physiological stress, allostatic load continues to exert a substantial direct effect on healthy ageing, highlighting the need for interventions targeting both stress reduction and social engagement.

The findings agree with the previous literature stating the buffering effect of social participation. Social interactions and community cohesion provide emotional and moral support that can mitigate the psychological distress associated with illness. Such supportive networks may enhance patients’ resilience and recovery, ultimately contributing to improved health outcomes [25,26].

Moreover, other studies showed that social connections serve as a stress buffer by reducing activity in stress-mediating neurobiological systems and reducing inflammation, thereby mitigating the physiological wear and tear associated with chronic stress [27,28]. Older adults who are socially engaged are more likely to sustain healthy behaviours, including regular physical activity and adherence to preventive care, both of which are established predictors of favourable ageing outcomes [29,30]. Finally, social participation fosters psychological well-being and resilience, which protect against the negative impacts of stress biology and support healthier ageing trajectories [31]. Taken together, these pathways highlight how social participation can attenuate, though not eliminate, the harmful effects of physiological stress, consistent with the partial mediation observed in this study.

Sociodemographic factors also played a significant role in later-life health outcomes. A higher level of education was associated with better health outcomes in later life, agreeing with previous research that showed higher educational attainment was associated with both higher baseline functioning and with later slower onset of cognitive declines [32].

Non-Black individuals exhibited poorer health outcomes compared to Black individuals. Although much of the ageing literature in the United States highlights poorer health trajectories among Black adults compared with White adults, the current results revealed the opposite pattern. Non-Black participants (comprising 86.1% of the sample and including White, Hispanic, and other groups) exhibited significantly worse healthy ageing than their Black counterparts. This finding may reflect the “mortality crossover” phenomenon, whereby Black adults who survive to older ages tend to be particularly resilient, often demonstrating lower mortality and better functional outcomes relative to non-Black peers [33]. Protective psychosocial resources, such as stronger community ties, religiosity, and social support, may further buffer the negative impact of chronic stress among older Black adults [34].

Additionally, the heterogeneity within the non-Black group, particularly the inclusion of Hispanic subgroups who have been shown to experience greater disability and frailty in later life, may contribute to these differences [35]. Thus, the current results underscore the importance of recognizing subgroup specific dynamics and resilience factors when examining ethnic inequalities in healthy ageing.

This study found a positive association between older age and achieving healthy ageing. This finding is consistent with mortality selection and selective attrition, whereby participants in poorer health are more likely to die or discontinue participation, leaving a progressively healthier cohort at later waves [36,37].

This study shows that both biological and social factors shape healthy ageing. Higher allostatic load directly predicted poorer ageing outcomes, while social participation partially attenuated this effect, indicating that engagement in social activities can buffer some negative impacts of chronic stress. Sociodemographic factors were also important. Non-Black individuals experienced worse ageing outcomes, whereas higher education was linked to healthier ageing trajectories. These findings highlight the cumulative and interconnected nature of physiological, social, and demographic influences on ageing.

This study has a few limitations. First, health behaviours were not included in the analysis. This was an intentional decision, as the focus was on examining baseline allostatic load and its longitudinal effects on social participation and healthy ageing. Incorporating health behaviours could have possibly altered the observed associations, either by attenuating or amplifying the effects of allostatic load. Second, socioeconomic status was captured only through education level. Although education is a strong and widely used indicator of socioeconomic status, it does not reflect other important dimensions such as income and wealth. These variables were excluded due to high levels of missing data.

## 5. Conclusions

This study advances the longitudinal literature on healthy ageing through the integration of biological and social factors. The findings highlight that social participation can shape the influence of chronic stress on later-life health. Addressing systemic racism and improving access to education could mitigate adverse ageing outcomes, while policies and interventions that reduce physiological stress and foster social engagement may support healthier and more equitable ageing trajectories. Future research should explore additional pathways and factors across diverse populations to inform strategies that promote healthy ageing, particularly by enhancing social participation and reducing stress to support resilience and overall well-being in older adults.

## Figures and Tables

**Table 1:** Participant characteristics for each wave included in the analysis (HRS-USA 2006–2010–2016) (*n* =14,538).

Year	Variables		Mean/Percentage
	Gender	Male	41.7%
		Female	58.3%
	Ethnicity	Black	13.9%
		Non-Black	86.1%
	Education	<High school	22.8%
		High school/equivalent	52.4%
		Some college	4.3%
		College and above	28.5%
2006			
	Age (mean)		68.7 (95%CI: 68.5–68.8)
	Marital status	Married	64.5%
		Unmarried/divorced	14.1%
		Widowed	21.4%
	Waist circumference	High risk	60.8%
	C-reactive protein	Elevated	19.2%
	Glycated hemoglobin	Diabetic	12.2%
	Blood pressure	Elevated	17.7%
	High density lipoprotein	Elevated	8.4%
2010			
	Healthy ageing		53.8%
	Social participation		78.3%
2016			
	Healthy ageing (outcome)		50.3%

**Table 2:** Path coefficient estimates using SEM (HRS-USA 2006–2010–2016) (*n=* 14,537).

Variables		Estimate	95% CI	*p*-Value
Allostatic Load 2006 (Latent Variable)				
	Waist circumference	0.95	(0.94, 0.96)	<0.001
	C-reactive protein	−0.90	(−0.90, −0.89)	<0.001
	High density lipoprotein	−0.37	(−0.38, −0.35)	<0.001
	Glycated hemoglobin	−0.35	(−0.38, −0.33)	<0.001
	Blood pressure	0.07	(0.05, 0.09)	<0.001
Social Participation 2010				
	Allostatic load 2006	−0.32	(−0.34, −0.30)	<0.001
Healthy ageing 2016				
2006	AL	−0.12	(−0.14, 0.11)	<0.001
	Education	0.18	(0.17, 0.19)	<0.001
	Age	0.36	(0.35, 0.37)	<0.001
	Marital status	0.02	(0.00, 0.03)	NS
	Gender	−0.01	(−0.02, 0.01)	NS
	Ethnicity	−0.11	(−0.12, −0.10)	<0.001
2010	Social Participation	0.05	(0.04, 0.06)	<0.001
	Healthy ageing	0.10	(0.08, 0.12)	<0.001
Model fit				
	RMSEA	0.54		<0.001
	CFI	0.05		
	TLI	0.00		

## Data Availability

The data that support the findings of this study are publicly available from the Health and Retirement Study (HRS) at https://hrs.isr.umich.edu/ and the RAND HRS Longitudinal File at https://www.rand.org/well-being/social-and-behavioral-policy/portfolios/aging-longevity/dataprod/hrs-data.html. No new data were generated in this study.
